# The Impact of Vitamin E and Other Fat-Soluble Vitamins on Alzheimer´s Disease

**DOI:** 10.3390/ijms17111785

**Published:** 2016-10-26

**Authors:** Marcus O. W. Grimm, Janine Mett, Tobias Hartmann

**Affiliations:** 1 Experimental Neurology, Saarland University, Kirrberger Str. 1, 66421 Homburg/Saar, Germany; janine.mett@uks.eu (J.M.); tobias.hartmann@mx.uni-saarland.de (T.H.); 2 Neurodegeneration and Neurobiology, Saarland University, Kirrberger Str. 1, 66421 Homburg/Saar, Germany; 3 Deutsches Institut für DemenzPrävention (DIDP), Saarland University, Kirrberger Str. 1, 66421 Homburg/Saar, Germany

**Keywords:** vitamin E, tocopherol, tocotrienol, Alzheimer´s disease, vitamin A, vitamin D, vitamin K, lipids

## Abstract

Alzheimer’s disease (AD) is the most common cause of dementia in the elderly population, currently affecting 46 million people worldwide. Histopathologically, the disease is characterized by the occurrence of extracellular amyloid plaques composed of aggregated amyloid-β (Aβ) peptides and intracellular neurofibrillary tangles containing the microtubule-associated protein tau. Aβ peptides are derived from the sequential processing of the amyloid precursor protein (APP) by enzymes called secretases, which are strongly influenced by the lipid environment. Several vitamins have been reported to be reduced in the plasma/serum of AD-affected individuals indicating they have an impact on AD pathogenesis. In this review we focus on vitamin E and the other lipophilic vitamins A, D, and K, and summarize the current knowledge about their status in AD patients, their impact on cognitive functions and AD risk, as well as their influence on the molecular mechanisms of AD. The vitamins might affect the generation and clearance of Aβ both by direct effects and indirectly by altering the cellular lipid homeostasis. Additionally, vitamins A, D, E, and K are reported to influence further mechanisms discussed to be involved in AD pathogenesis, e.g., Aβ-aggregation, Aβ-induced neurotoxicity, oxidative stress, and inflammatory processes, as summarized in this article.

## 1. Introduction

**Figure 1:**
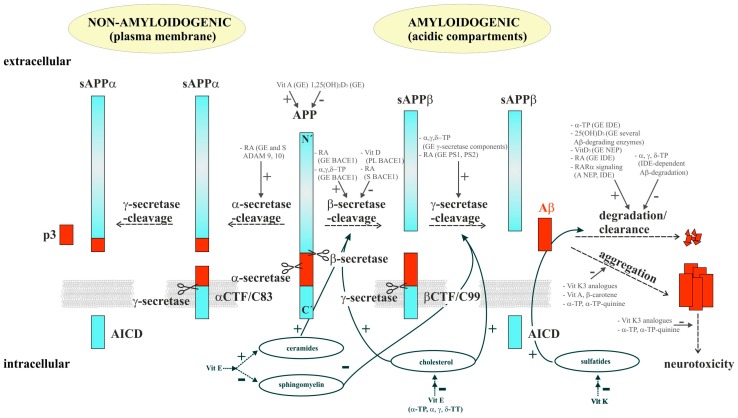
Proteolytic processing of the amyloid precursor protein (APP) and its modulation by vitamins A, D, E, and K. APP can be cleaved in two different processing pathways. In the amyloidogenic processing pathway (**right**) APP is first cleaved by β-secretase BACE1 (β-site APP cleaving enzyme 1) generating soluble sAPPβ (soluble β-secreted APP) and the C-terminal anchored fragment C99/βCTF (β-cleaved C-terminal fragment), which is further cleaved by the γ-secretase complex to release amyloid-β (Aβ) peptides. Aβ peptides can be degraded by several processes or aggregate to build up neurotoxic amyloid plaques. In the non-amyloidogenic pathway (**left**) APP is first cleaved within the Aβ domain by α-secretases (members of the ADAM (a disintegrin and metalloprotease) protein family) leading to the release of soluble sAPPα (soluble α-secreted APP) and the generation of the C-terminal membrane-tethered fragment C83/α-CTF (α-cleaved C-terminal fragment), which is also cleaved by the γ-secretase complex to release the non-toxic peptide p3. In both processing pathways the APP intracellular domain (AICD) is liberated into the cytosol. GE = gene expression; S = sorting; A = activity; RA = retinoic acid; Vit = vitamin; TP = tocopherol; PL = protein level; TT = tocotrienol; PS1 = presenilin 1; PS2 = presenilin 2; IDE = insulin degrading enzyme; NEP = neprilysin. The effects of vitamins on AD-relevant mechanisms are indicated by solid arrows, the impact of vitamins on lipid homeostasis is delineated by dashed arrows. + = increase; − = decrease.

Alzheimer’s disease (AD) is the most common cause of dementia among neurodegenerative diseases in the elderly population, clinically characterized in patients by an inexorable progression leading to memory loss and cognitive decline [1,2]. Currently, 46 million people worldwide are estimated to be affected by AD and its incidence will dramatically increase due to increasing life expectancy and average age (the number of affected individuals is estimated to be doubled every 20 years), emphasizing AD as a major public health concern [3,4]. The brain tissue of AD-affected individuals shows a significant loss of synapses and neurons resulting in a strong hippocampal and cortical atrophy [5,6,7,8]. The characteristic histopathological hallmarks of AD are extracellular neuritic plaques and intracellular neurofibrillary tangles in vulnerable brain regions, like the hippocampus and cortex [9]. The neuritic plaques are mainly composed of amyloid-β peptides (Aβ), whereas intracellular neurofibrillary tangles consist of an abnormally phosphorylated form of the microtubule-associated protein tau [10,11]. Aβ peptides are generated by sequential proteolytic processing of the amyloid precursor protein (APP), a ubiquitously-expressed type I transmembrane protein [12,13]. For the generation of Aβ peptides APP is first cleaved by β-secretase BACE1 (β-site APP cleaving enzyme 1) in acidic intracellular compartments generating soluble sAPPβ and a C-terminal membrane-tethered fragment called C99/βCTF (β-cleaved C-terminal fragment), which is further cleaved by the γ-secretase complex to release Aβ peptides (Figure 1). The γ-secretase complex consists of at least four proteins, presenilin (PS) 1 or 2 as catalytically-active components, nicastrin, anterior pharynx defective 1 (Aph1) a or b, and presenilin enhancer 2 (PEN-2).

The severe accumulation of Aβ within brain tissue, starting years or even decades prior to the first symptoms, is considered as one of the major factors of AD pathogenesis. It might be caused by an imbalance between the production and clearance of the peptide [14,15]. Especially, an increase in Aβ42, the major Aβ species found in amyloid plaques, is reported to trigger the disease process [16,17]. However, in addition to the amyloid hypothesis, several other mechanisms, like inflammatory processes, tau pathology, a disruption of calcium homeostasis, and membrane integrity, cholinergic, and mitochondrial dysfunction and increased oxidative stress play an important role in AD, as well [18,19,20]. In addition to the amyloidogenic processing of APP by β- and γ-secretase APP can be cleaved at the cell surface in a non-amyloidogenic pathway preventing the generation of Aβ peptides. In this case APP is first cleaved within the Aβ domain by α-secretases, identified as members of the ADAM (a disintegrin and metalloprotease) protein family, generating soluble sAPPα and the C-terminal membrane-tethered fragment C83/α-CTF. In analogy to C99/β-CTF, C83/α-CTF is also cleaved by the γ-secretase complex to release the non-toxic peptide p3. In both processing pathways the γ-secretase-dependent cleavage of C83/α-CTF and C99/β-CTF results in the intracellular liberation of AICD (APP intracellular domain) (Figure 1), which is believed to translocate into the nucleus and to regulate the expression of several target genes [21]. Amyloidogenic, as well as non-amyloidogenic, processing of APP belong to the reaction of regulated intramembrane proteolysis (RIP), characterized by a primary cut outside the membrane followed by a second cleavage within the membrane-spanning domain [22]. The γ-secretase complex responsible for the second cut of APP-RIP belongs to the intramembrane-cleaving proteases that can function within the hydrophobic lipid environment of cellular membranes. All components of the γ-secretase complex are transmembrane proteins, further emphasizing the importance of lipids in APP processing and AD pathogenesis. Several lipids, including cholesterol, the polyunsaturated fatty acid (PUFA) docosahexaenoic acid (DHA), sphingomyelin, gangliosides, plasmalogens, and *trans* fatty acids, have been shown to alter APP processing [23,24,25,26,27,28,29]. Furthermore, hypercholesterolemia is believed to be a risk factor to develop AD [26,30]. Lipids, especially PUFAs, are very susceptible to reactive oxygen species (ROS) and lipid peroxidation, resulting in oxidative stress, known to be involved in AD pathogenesis [31]. Notably, recently it has been shown that different oxidation products of DHA increase amyloidogenic processing of APP, whereas unoxidized DHA is known to decrease Aβ levels [24,32], emphasizing the need to prevent DHA and other lipids from oxidation in nutritional approaches. Due to its anti-oxidative activity vitamin E is especially believed to be beneficial for AD. Vitamin E belongs to the fat-soluble vitamins, like vitamin A, D and K. A large portion of the elderly population is inadequately supplied with several vitamins known to be necessary for proper brain function and several hypovitaminosis have been linked to an enhanced AD-risk [33]. In the following review we focus on the impact of vitamin E and the other lipophilic vitamins A, D, and K on AD incidence and pathogenesis. As summarized below, the impact of vitamins on AD might be based on both direct effects on AD-relevant mechanisms and their interference with cellular lipid homeostasis. 

## 2. Vitamin A

Vitamin A and its derivatives, the retinoids, are involved in several important cellular processes in the brain, including neuronal differentiation, neurotransmitter release, and long-term potentiation. They possess anti-oxidative properties and the ability to regulate gene expression by interacting with the retinoic acid receptors (RARs) and the retinoid X receptors (RXRs) acting as transcription factors [34,35].

Significantly-lowered serum and plasma concentrations of vitamin A and the provitamin A β-carotene have been observed in AD patients [36,37,38] and enhanced β-carotene plasma levels have been found to be associated with better cognitive performances in the elderly [39]. Interestingly, retinoid acid synthesis is repressed in response to Aβ peptides by a RARα-dependent mechanism [40], indicating vitamin A production to be reduced in tissues containing high amounts of Aβ peptides, like brain tissue affected by AD.

Vitamin A deficiency results in an enhanced Aβ deposition in the brain tissue and in cerebral blood vessels of adult rats [41]. In a recent study Aβ production was also shown to be increased in the brain parenchyma of a hypovitaminosis mouse model along with a reduction of sAPPα content indicating a shift from non-amyloidogenic to amyloidogenic APP processing [42]. In a double-transgenic AD mouse model a robust decrease of cerebral Aβ accumulation along with improved cognitive functions was observed after treatment with all-*trans* retinoic acid over eight weeks [43]. Similar effects on cognition and Aβ plaque load have been observed in mice with streptozotocin-induced dementia after supplementation with all-*trans* retinoic acid. These animals also display a restored acetylcholinesterase activity, attenuated oxidative alterations and a reduced content of myeloperoxidase, a marker of inflammation [44]. In line with this, treatment of APP/tau-double transgenic mice with a RARα agonist leads to a reduction of Aβ plaque load and enhanced cognitive performances. In this study an activation of the Aβ-degrading enzymes neprilysin (NEP) and insulin-degrading enzyme (IDE) has been demonstrated in microglia after activation of RARα signaling, which might lead to the reduced Aβ pathology in animals treated with the RARα agonist [40]. In addition to the cerebral Aβ content tau pathology and, thus, a further pathological hallmark of AD, might be affected by vitamin A. Previously fewer and smaller tau aggregates have been observed in the brain tissue of APP/PS1/tau-transgenic mice supplemented with all-*trans* retinoic acid. This effect might result from a down-regulation of the cyclin-dependent kinase 5 (Cdk5) and the glycogen synthase kinase 3β (GSK3β) [45]. In agreement with these data, a prevented tau phosphorylation has been demonstrated in APP/tau-double transgenic mice medicated with a RARα agonist, as well as in APP/PS1 transgenic mice treated with retinoic acid [40,43]. However, so far there are no trials analyzing the impact of vitamin A supplementation on the progression of AD in humans. 

The effects of vitamin A and its derivatives on the cerebral Aβ level might be, at least partially, explained by a retinoid-dependent transcriptional regulation of AD-relevant genes, including those encoding for APP, BACE1, PS1, PS2, ADAM 9, ADAM 10, and IDE [46,47,48,49,50,51,52]. In addition to these transcriptional effects vitamin A seems to additionally affect APP processing by altering the intracellular sorting of the secretases. All-*trans* retinoic acid has been shown to increase the translocation of ADAM9, ADAM10, and BACE1 to the cellular membrane leading to enhanced non-amyloidogenic APP processing and impaired β-secretase-dependent APP cleavage [51]. Additionally, vitamin A and β-carotene were found to negatively affect the oligomerization of Aβ and the stability of preformed Aβ fibrils in vitro, possibly via binding to the C-terminal region of the peptide [53,54]. 

## 3. Vitamin D

Vitamin D_3_ (cholecalciferol) belongs to the calciferols, a group of fat-soluble secosterols, including vitamin D_2_ (ergocalciferol). Vitamin D is important for physiological functioning and protection of the central nervous system (CNS). Anti-oxidative, anti-ischemic and anti-inflammatory actions of vitamin D_3_ have been described as well as a link to neurotransmitter levels. While vitamin D_2_ is largely found in food, vitamin D_3_ is mainly synthesized from 7-dehydrocholesterol in the human skin upon exposure to sunlight (ultraviolet B, 297–315 nm) and, to a lesser extent, also taken up with diet. 1α,25-(OH)_2_D_3_ (calcitriol), biologically the most active form of vitamin D_3_, is generated by hydroxylation of cholecalciferol in the liver and kidney. 1α,25-(OH)_2_D_3_ is able to modulate gene expression via binding to the nuclear vitamin D receptor (VDR). The VDR forms a complex with the retinoid X receptor (RXR), which influences transcription by interacting with the vitamin D response element (VDRE) [55,56].

Several studies reported a reduced vitamin D_3_ concentration in the serum/plasma of patients suffering from all cause dementia and AD [33,57,58]. Additionally, low serum vitamin D_3_ levels were found to be associated with an enhanced risk of cognitive decline in general and in AD [59,60,61,62,63]. In contrast, elevated 25-(OH)D_3_ plasma/serum levels have been linked to increased cognitive function and greater volumetric measures of several brain structures typically affected by AD [64,65]. A link between vitamins D and AD risk is further given by the observation that several VDR polymorphisms are associated with AD susceptibility [66,67,68,69]. There are some trials analyzing the potential benefit of vitamin D supplementation for patients already suffering from AD. Annweiler et al. demonstrated the supplementation of vitamin D_3_ improved cognition and memory in patients with moderate AD receiving memantine [70]. This might be based on a synergistic neuroprotective effect of memantine plus vitamin D, as illustrated by the reduction of Aβ-induced axonal degeneration in the presence of these compounds [71]. The authors suggested the combination of memantine and vitamin D_3_ to represent a new multi-target therapeutic class for AD treatment [70,72]. In contrast to vitamin D_3_, the supplementation of vitamin D_2_ seems to have no impact on the cognitive performance of nursing home residents and persons with mild to moderate severe AD [73,74].

Vitamin D affects several mechanisms of AD pathogenesis, including the production, clearance, phagocytosis, and enzymatic degradation of Aβ peptides, as well as tau phosphorylation [75]. Rodents fed with vitamin D_3_-enriched diets show a significant reduction of brain Aβ burden along with improved cognitive performances [76,77,78]. Inversely, we and others observed an increase of the Aβ40 and Aβ42 content in the brain tissue of vitamin D-deficient animals [78,79]. This is in line with the observation that vitamin D deficiency strengthens the spatial learning deficits of rats after intracerebroventricular Aβ42-injection. However, in this study additional supplementation of 1,25(OH)_2_D_3_ did not significantly improve the spatial performance of rats with normal vitamin D levels [80]. 

The influence of vitamin D on the cerebral Aβ levels might be based on an increased brain-to-blood efflux transport of the peptide at the blood-brain barrier and a stimulation of microglial Aβ phagocytosis [77,81,82]. Moreover, an elevation of NEP, one of the major Aβ-degrading enzymes, along with a reduction in BACE1 protein content, has been shown in the brain tissue of aged rats after dietary supplementation of vitamin D_3_ [77]. In line with this, we found a significantly increased protein level and activity of BACE1 combined with a decreased expression and activity of NEP in the brain tissue of vitamin D-deficient mice [79]. An impact of vitamin D on Aβ degradation is further strengthened by the transcriptional up-regulation of several Aβ-degrading enzymes and, hence, total Aβ degradation in neuroblastoma cells treated with 25-(OH)D_3_ [79]. In another study,1α,25-(OH)_2_D_3_ has been found to inhibit APP promoter activity in a neuroblastoma cell line, indicating Aβ secretion to be reduced in the presence of this vitamin D metabolite due to a decreased gene expression of its precursor protein [69]. Additionally, vitamin D might affect tau phosphorylation, as previously published by Cheng et al. In this study the combination of vitamin D and resveratrol has been found to reverse the Aβ25-35-induced cytotoxicity and tau phosphorylation in SH-SY5Y cells [83].

## 4. Vitamin E

The E-vitamins are a group of structurally-related, lipid-soluble antioxidants found in all cellular membranes. They are included in several vegetable oils and essential for humans and animals. The vitamin E family mainly includes eight compounds, α-, β-, γ-, and δ-tocopherols and tocotrienols. α-Tocopherol is the most common vitamin E form in human tissues and the major E-vitamin used in supplements. Due to the anti-oxidative potential of vitamin E protecting lipids from peroxidation in membranes, vitamin E supplementation has been suggested to be beneficial in AD. In addition to their anti-oxidative properties, molecules of the vitamin E family exert neuroprotective, anti-inflammatory, and hypocholesterolemic activities [84,85,86]. Vitamin E is also able to modulate gene expression by influencing several transcriptional pathways, including the PPARγ (peroxisome proliferator-activated receptor γ)- and NF-κB (nuclear factor-κB)-pathways [87,88].

In the plasma of patients with AD and mild cognitive impairment (MCI), significantly lowered vitamin E levels have been found [36,37,89,90]. Inversely, higher plasma vitamin E concentrations and an enhanced dietary intake of vitamin E or α-tocopherol equivalents are associated with a reduced AD risk [91,92,93]. Recently, an association between an enhanced γ-tocopherol level and lowered AD neuropathology in human post mortem AD-brain tissue was reported, while α-tocopherol is associated with a higher Aβ load when γ-tocopherol levels are low [94]. 

Several studies analyzed the impact of vitamin E supplementation on AD progression leading to inconsistent results. While a reduction of the need for care and of disease progression for AD-patients treated with 2000 IU/day α-tocopherol was reported in some trials [95,96], other authors found vitamin E supplementation to have no beneficial effect, or to result in an even more rapid cognitive decline in patients with MCI or AD [97,98]. In this context it should be mentioned that high dosage vitamin E supplements might increase all-cause mortality, as reported by Miller et al., leading the authors to conclude that dosages of more than 400 IU vitamin E/day should be avoided [99].

The potential beneficial effect of vitamin E supplementation has also been analyzed in transgenic AD mouse models. Vitamin E supplementation leads to a reduced cerebral Aβ content in young, but not in aged, APP-transgenic mice [100]. In another study dietary supplementation with α-tocopherol, *N*-acetylcysteine, and α-lipoic acid attenuated age-related alterations in Aβ metabolism in aged rat brains and prevented deficits in learning and memory functions [101,102]. Similar effects have been observed for α-tocopherol quinine, an oxidative metabolite of α-tocopherol. Oral administration of this compound ameliorates memory impairment in APP/PS1 double transgenic mice and reduces the cerebral levels of Aβ oligomers. Additionally, it decreases oxidative stress and the production of inflammatory mediators in these animals [103]. α-tocopherol and α-tocopherol quinine have also been found to negatively affect Aβ aggregation, Aβ-induced toxicity, inflammatory processes, the generation of ROS, and the oxidation of lipids in cultured cells [103,104,105,106,107]. The fact that only 1% of DHA-lipid peroxidation products is sufficient to reverse the Aβ-lowering effect of unoxidized DHA emphasizes the relevance of preventing lipids from oxidation [32]. In addition, chronic α-tocopherol depletion, by knocking out the α-tocopherol transfer protein, enhances Aβ deposition in the brain of APP-transgenic mice with amelioration of the effects after α-tocopherol reception [108]. This might be due to a reduced gene expression and protein level of IDE resulting in an impaired enzymatic degradation of Aβ peptides in the α-tocopherol transfer protein-deficient mice [109]. An impact of chronic vitamin E deficiency on several genes encoding for proteins directly or indirectly involved in Aβ clearance has been confirmed in a further study analyzing gene expression in the rat hippocampus [110]. In our recently published study we tested the effect of α-, γ-, and δ-tocopherol on Aβ production and degradation in neuroblastoma cell lines. Surprisingly, all tested tocopherol species were associated with increased Aβ secretions. These effects were found to be based on an increased gene expression of the β- and γ-secretase components along with an inhibition of Aβ-degradation [111]. Accordingly, under some conditions this amyloidogenic potential of some vitamin E molecules might attenuate the indisputable positive anti-oxidative effect of vitamin E as Aβ is known to increase oxidative stress [112]. 

The interaction between different molecules or pathways involved in AD pathogenesis is further illustrated by the hypocholesterolemic effect of vitamin E. Recently it has been shown that vitamin E decreases the cholesterol level by affecting the sterol regulatory element binding protein (SREBP)/SCAP (SREBP cleavage-activating protein) system, one of the main systems controlling the cellular cholesterol level. When cholesterol levels are low SREBP is transported from the endoplasmic reticulum to the Golgi-apparatus where the N-terminal domain of SREBP is shed of, which translocates to the nucleus to up-regulate the expression of genes involved in cholesterol de novo synthesis, including hydroxymethylglutaryl-CoA reductase (HMGCR) [113,114]. δ-Tocotrienol blocks processing of SREBP and stimulates HMGCR degradation, γ-tocotrienol has been shown to primarily enhance HMGCR degradation [115], whereas α-tocotrienol and all tocopherols show no effect on SREBP processing or HMGCR degradation. However, α-tocopherol and α-tocotrienol decrease activated nuclear SREBP in other studies resulting in the reduction of genes involved in cholesterol de novo synthesis [116,117]. The majority of cell culture studies found a reduced cellular cholesterol content to be associated with decreased Aβ production while an increased cholesterol level has the opposite effect [118,119,120]. A strong correlation between hypercholesterolemia and an enhanced Aβ level has also been observed in several animal models [120,121,122]. It is well established that these effects are based on a direct stimulation of β- and γ-secretase activity by cholesterol [23,123,124]. As amyloidogenic APP-processing is mainly localized in cholesterol-dependent lipid raft microdomains, the generation of Aβ correlates with the integrity of these membrane structures. Cholesterol depletion reduces amyloidogenic APP-processing due to a reduced association of APP, BACE1, and the γ-secretase as a result of lipid raft disruption. In contrast, an increase of the membrane cholesterol level leads to a higher lipid raft content of the membranes and, thus, to enhanced Aβ generation [118,125,126]. High cellular cholesterol levels additionally stimulate the internalization of APP leading to Aβ overproduction in acidic intracellular compartments [127]. Contrarily, APP is primarily localized at the cell surface in cholesterol-depleted cells, resulting in an enhanced α-secretase-dependent APP processing [128]. In addition to these effects on the proteolytic processing of APP, a promotion of Aβ aggregation and Aβ-induced toxicity by cholesterol has been reported [129,130,131].

The cellular lipid homeostasis is further altered by vitamin E because of its stimulating effect on phospholipase A2 (PLA2) activity resulting in an increased release of arachidonate [132]. As arachidoneic acid is known to activate the neutral sphingomyelinase (nSMase) [133], higher vitamin E levels might indirectly result in an increased cellular ceramide content along with reduced levels of sphingomyelin. Enhancing the levels of pro-apoptotic and neurotoxic ceramides [134,135] by ceramide- or nSMase-supplementation to cultured cells stimulates amyloidogenic APP processing and, hence, results in increased Aβ generation. The underlying mechanism has been identified as a post-translational stabilization of BACE1 by ceramides due to enhanced acetylation of the protein [136,137]. In contrast to ceramides, sphingomyelin has been demonstrated to inhibit Aβ production. Accumulation of sphingomyelin by either direct supplementation or the inhibition of nSMase in cultured cells results in a significant reduction of Aβ peptides due to an inhibition of γ-secretase-dependent APP cleavage [25]. Thus, the activation of PLA2-activity by vitamin E might result in an enhanced production of Aβ peptides due to an alteration of the cellular ceramide/sphingomyelin ratio. This is strengthened by the observation that genetic deficiency or reduction of GIVA-PLA2 (group IV isoform of PLA2) protects APP transgenic mice against Aβ-induced cognitive deficits and premature mortality [138].

Similar to vitamins A and D, vitamin E and its derivatives have also been reported to influence tau pathology. The treatment of cultured neurons with trolox, a water-soluble analog of vitamin E, prevents Aβ-induced tau hyperphosphorylation, as previously described by Giraldo et al. [139]. In contrast, in another study, vitamin E precludes ROS generation and apoptosis, but did not affect tau phosphorylation in differentiated SH-SY5Y cells treated with Aβ peptides [140]. An effect of vitamin E on tau-pathology has also been analyzed in vivo by using different animal models. The supplementation of tau transgenic mice with α-tocopherol results in a suppressed/delayed development of tau pathology along with improved health and reduced motor weakness [141]. Additionally, tau-induced neurodegeneration in *Drosophila* has been demonstrated to be reduced after treatment of adult flies with vitamin E [142]. 

To summarize, vitamin E regulates and interferes with several molecular mechanisms which have been shown to be highly linked to AD. Interestingly, vitamin E is not only associated with beneficial cellular changes in respect to AD. For example, the advantageous cholesterol-lowering and anti-oxidative effects of vitamin E are accompanied by an increased cellular ceramide/sphingomyelin ratio, which is discussed to be unfavorable for AD pathogenesis. From this point of view one might speculate that not all patients suffering from AD or MCI profit from vitamin E supplementation or that even “responders” and “non-responders” exist. This might be a possible explanation for the heterogeneous results of nutritional approaches analyzing the impact of vitamin E treatment on AD progression.

## 5. Vitamin K

Vitamin K is a group of fat-soluble molecules, including the naturally-occurring vitamin K1 (phylloquinone) and vitamin K2 (menaquinone), as well as the synthetic vitamin K3 (menadione). While vitamin K1 is mainly found in green vegetables and olive oil, vitamin K2 is present in small amounts in chicken, eggs, and butter. In the CNS vitamin K occurs predominantly as menaquinone-4 and regulates the activity of proteins involved in cell growth, myelination, mitogenesis, chemotaxis, and neuroprotection [143,144]. 

In analogy to vitamins A, D, and E, the dietary intake of phylloquinone and, hence, the serum vitamin K concentration, is reported to be decreased in persons suffering from AD [145,146,147]. A possible role of vitamin K in AD-pathogenesis is further given by the discovery of a positive correlation between the serum vitamin K level and the cognitive functions of AD patients [147,148]. Additionally, the use of vitamin K antagonists as anticoagulant medications is associated with a more frequent cognitive impairment among geriatric patients [149]. 

A similar association between vitamin K and cognition has been observed in rats, as reported by Carrié et al. In this study a diet low in phylloquinone resulted in increased cognitive deficits in aged, but not in young, rats [150]. In a recent in vitro study vitamin K3 analogs have been found to effectively inhibit Aβ aggregation and to protect neuroblastoma cells from Aβ-induced toxicity. These results indicate that vitamin K might be an effective anti-amyloidogenic drug [151]. However, so far there are no further data available regarding the impact of vitamin K on the pathological mechanisms of AD.

Interestingly, vitamin K also modulates brain sphingolipid metabolism by stimulating the activity of serine palmitoyl-CoA transferase (SPT) and cerebrosidesulfotransferase (CST). SPT initiates sphingolipid biosynthesis by catalyzing the condensation of palmitoyl-CoA and L-serine to 3-ketosphinganin, which is further metabolized to ceramide, an important branching point within the sphingolipid metabolism pathways. CST is one of the enzymes involved in the conversion of ceramides to sulfatides. Accordingly, vitamin K deficiency leads to significantly reduced cerebral sulfatide levels in rodents, while there is an opposite effect in vitamin K-supplemented animals [152,153]. Sulfatides, which seem to be decreased in brain tissue and cerebrospinal fluid (CSF) of AD-patients [154,155,156,157], have been reported to be associated with a strong reduction of Aβ peptides in the media of cultured cells. This might be due to a facilitated Aβ clearance through an endocytotic pathway as response to enhanced cellular sulfatide concentrations. Vitamin K-deficient rats further show enhanced concentrations of ceramides in the hippocampus and lower ganglioside levels in the pons medulla and midbrain [150]. Overall these data indicate vitamin K deficiency to be accompanied by alterations in sphingolipid homeostasis, which are unfavorable in respect to the mechanisms involved in the production and clearance of Aβ peptides.

## 6. Conclusions

As summarized in Table 1, there is a strong link between fat-soluble vitamins and AD. A deficiency of vitamins A, D, E, and K have been reported to be tightly associated with AD. In contrast, enhanced serum/plasma levels of these vitamins have been linked to increased cognitive functions. Vitamins D and E hypovitaminosis seem to be a risk factor for the development of AD and there are some indications that the supplementation of these vitamins might be beneficial in halting AD progression.

The fact that vitamins A, D, E, and K deficiency results in increased cerebral Aβ levels and/or weakened cognitive performances in animal models while the supplementation of vitamins A, D, and E reduces Aβ plaque load strengthens the assumption that the supplementation of these compounds might be beneficial. However, especially for vitamin E, supplementation may result in some unfavourable effects as, for example, an Aβ-increasing potential and an even more rapid cognitive decline in AD- and MCI-patients have been reported in some studies.

In conclusion, further studies, especially large trials analyzing the effectiveness of the different vitamins in AD-patients and their influence on the molecular mechanisms of the disease, are required to evaluate the potential of these compounds in the prevention and therapy of AD.

## Figures and Tables

**Table 1:** Summary of the impact of vitamins A, D, E, and K on Alzheimer’s disease (AD) risk, cognitive functions, and the molecular mechanisms of AD. The presented data are derived from in vitro experiments using, e.g., cell culture models, in vivo models, such as transgenic animals, and the analysis of AD patients, but not from clinical experiments. MCI, mild cognitive impairment; APP, amyloid precursor protein; ADAM 9, a disintegrin and metalloprotease 9; ADAM10, a disintegrin and metalloprotease 10; BACE1, β-site APP cleaving enzyme 1; PS1, presenilin 1; PS2, presenilin 2; IDE, insulin degrading enzyme; →, effect; ↑, increase; ↓, decrease.

Effect/Changes	Vitamin A	Vitamin D	Vitamin E	Vitamin K
Vitamin level in serum/plasma of AD/MCI patients	↓ Vitamin A and provitamin A levels in serum/plasma of AD patients [36,37,38]	↓ Vitamin D concentration in serum/plasma of patients suffering from all cause dementia and AD [33,57,58]	↓ Vitamin E levels in plasma of patients suffering from AD or MCI [36,37,89]	↓ Serum vitamin K concentration in persons suffering from AD [145,146,147]
Effect of serum/plasma vitamin level on cognition/AD risk in humans	↑ β-carotene plasma levels →↑ Cognitive performances in the elderly [39]	↓ Serum vitamin D levels →↑ Risk of cognitive decline and AD [59,60] ↑ 25-(OH)D_3_ plasma/serum levels →↑ Cognitive function, ↑ volumetric measures of brain structures affected by AD [64,65]	↑ Plasma vitamin E levels/↑ intake of vitamin E or α-tocopherol equivalents →↓ Risk of AD [91,92,93]	
Effect of serum/plasma vitamin level/vitamin supplementation on AD progression in humans		Supplementation of vitamin D_3_ →↑ Cognition and memory in patients with moderate AD receiving memantine [70,72]	Inconsistent results [95,96,97,98]	Positive correlation between serum vitamin K level and cognition of AD patients [147,148]
Effect of vitamin-supplementation on AD pathology/cognition in animal models	Treatment with retinoic acid →↓ Cerebral Aβ deposition, ↓ tau phosphorylation, ↑ cognitive functions [43,44] Treatment with all-*trans* retinoic acid →↓ Tau aggregation [45]	Vitamin D-enriched diet →↓ Brain Aβ burden, ↑ cognitive performances [76,77,78]	Vitamin E supplementation →↓ Cerebral Aβ-content in young APP-transgenic mice [100] →↓ Tau-induced neurodegeneration in *Drosophila* [142] α-tocopherol supplementation →↓ of age-related alterations in Aβ metabolism, ↓ deficits in learning and memory functions [101,102] →↓ Tau pathology [141] α-tocopherol quinine supplementation →↓ Memory impairment, ↓ cerebral levels of Aβ oligomers [103]	
Effect of vitamin-deficiency on AD pathology/cognition in animal models	Vitamin A deficiency →↑ cerebral Aβ deposition [41] →↑ cerebral Aβ production, ↓ cerebral sAPPα level [42]	Vitamin D deficiency →↑ Cerebral Aβ40 and Aβ42 levels [78,79] →↑ Spatial learning deficits [80]	α-tocopherol deficiency →↑ Cerebral Aβ-deposition [108]	Phylloquinone deficiency →↓ Cognitive functions in aged rats [150]
Effect of vitamin on AD-relevant molecular mechanisms	Retinoid acid →↑ Gene expression of APP, ADAM 9, ADAM10, BACE1, PS1, PS2, and IDE [47,48,49,50] →↑ Translocation of ADAM9, ADAM10, and BACE1 to the cellular membrane [51] vitamin A and β-carotene →↓ Aβ oligomerization, ↓ stability of Aβ fibrils [53,54]	Vitamin D →↑ Aβ clearance across the blood brain barrier, ↑ microglial Aβ-phagocytosis [77,81,82] →↑ NEP protein level, ↓ BACE1 protein level [77] →↓ Tau phosphorylation, ↓ Aβ-induced toxicity [83] 25-(OH)D_3_ →↑ Gene expression of Aβ-degrading enzymes [79] 1α,25-(OH)_2_D_3_ →↓ APP promoter activity [69]	α-, γ-, and δ-tocopherol →↑ Gene expression of the β- and γ-secretase components, ↓ Aβ-degradation [111] α-tocopherol, α-tocopherol quinine →↓ Aβaggregation, ↓ Aβ-induced toxicity [103,105,106,107] trolox (water-soluble vitamin E analog) →↓ Aβ-induced tau hyperphosphorylation [139]	Vitamin K3 analogues →↓ Aβ-aggregation, ↓ Aβ-induced toxicity [151]
